# Hemodialysis Patients May Benefit from Cholecalciferol Treatment Targeting High Level of 25(OH)D

**DOI:** 10.3390/medicina60111831

**Published:** 2024-11-07

**Authors:** Agnieszka Tarasewicz, Małgorzata Dąbrowska, Michał Komorniczak, Agnieszka Zakrzewska, Bogdan Biedunkiewicz, Sylwia Małgorzewicz, Magdalena Jankowska, Katarzyna Jasiulewicz, Natalia Płonka, Alicja Dębska-Ślizień, Leszek Tylicki

**Affiliations:** 1 Department of Nephrology, Transplantology and Internal Medicine, Medical University of Gdańsk, 80-210 Gdańsk, Poland; michal.komorniczak@gumed.edu.pl (M.K.); agnieszka.zakrzewska@gumed.edu.pl (A.Z.); bogdan.biedunkiewicz@gumed.edu.pl (B.B.); magdalena.jankowska@gumed.edu.pl (M.J.); katarzyna.jasiulewicz@gumed.edu.pl (K.J.); natalia.plonka@gumed.edu.pl (N.P.); adeb@gumed.edu.pl (A.D.-Ś.); leszek.tylicki@gumed.edu.pl (L.T.); 2 Central Clinical Laboratory, University Clinical Center, 80-952 Gdańsk, Poland; mdabrowska@uck.gda.pl; 3 Department of Clinical Nutrition, Medical University of Gdańsk, 80-210 Gdańsk, Poland; sylwia.malgorzewicz@gumed.edu.pl

**Keywords:** cholecalciferol, chronic kidney disease–mineral and bone disorder, hemodialysis, parathormone, vitamin D, 25-hydroxycholecalciferol

## Abstract

(1) *Background and Objectives*: Vitamin D is implicated in the pathogenesis of Chronic Kidney Disease—Mineral and Bone Disorder (CKD-MBD) in hemodialysis (HD) patients, including the development of secondary hyperparathyroidism (SHP). While cholecalciferol supplementation is recommended for vitamin D deficiency correction, its impact on CKD-MBD remains inconsistent. The aim of this observational prospective study was to assess the effect of cholecalciferol in achieving high–normal serum 25-hydroxycholecalciferol (25(OH)D > 75 ng/mL) levels and its impact on CKD-MBD biochemical markers, including 1,25-dihydroxycholecalciferol (1,25(OH)_2_D) and parathormone (PTH) in HD patients. The study also evaluated the maintenance dosage required to sustain 25(OH)D levels within the 50–75 ng/mL range. (2) *Materials and Methods*: A total of 22 HD patients with baseline 25(OH)D levels 30–50 ng/mL received cholecalciferol (70,000 IU/week) to achieve the target serum 25(OH)D > 75 ng/mL. Baseline data on calcium, phosphate, 1–84 PTH, 25(OH)D, and 1,25(OH)_2_D serum levels were compared with the data when 25(OH)D > 75 ng/mL was targeted or when the highest 25(OH)D levels were noted. (3) *Results*: Cholecalciferol significantly improved vitamin D status in HD patients, with 73% reaching the target 25(OH)D level >75 ng/mL in a median time of 7.5 weeks, with a median total dose of 525,000 IU. This was associated with a significant rise in 1,25(OH)_2_D, decrease in 1–84 PTH, and no significant effect on calcium and phosphate levels. The median maintenance dose of cholecalciferol was established at 30,000 IU/week. (4) *Conclusions*: The findings support the use of cholecalciferol targeting high normal 25(OH)D levels to improve biochemical markers of CKD-MBD in HD patients.

## 1. Introduction

Chronic Kidney Disease—Mineral and Bone Disorder (CKD-MBD) is a systemic disorder of mineral and bone metabolism that occurs as a complication of chronic kidney disease (CKD). It encompasses a spectrum of abnormalities related to calcium, phosphorus, parathyroid hormone (PTH), and vitamin D metabolism, abnormalities in bone structure and turnover, as well as vascular or other soft tissue calcification [1]. One of the early manifestations of CKD-MBD is an increased secretion of PTH, known as secondary hyperparathyroidism (SHP). The rise in PTH levels in CKD has been primarily induced by a progressive decline in the synthesis of the active form of vitamin D, 1,25-dihydroxycholecalciferol (1,25(OH)_2_D, calcitriol), hyperphosphatemia, and hypocalcemia. However, even in hemodialysis (HD) patients, 1,25(OH)_2_D decrease is partially a consequence of native 25-hydroxycholecalciferol (25(OH)D, calcidiol) deficiency, rather than only inadequate activation of vitamin D by renal and extra-renal 1-α-hydroxylase. Current guidelines for the prevention and treatment of vitamin D deficiency in CKD patients reveal a great heterogeneity [2,3].

According to current KDIGO (Kidney Disease: Improving Global Outcomes) guidelines, in patients with CKD on dialysis, it is suggested that 25(OH)D levels should be measured and vitamin D deficiency and insufficiency corrected using treatment strategies recommended for the general population. In the context of SHP, the topic of vitamin D supplementation is only raised in non-dialyzed CKD patients with persistently rising or elevated PTH, where the evaluation should also address vitamin D deficiency [4]. The use of native vitamin D in HD patients is still controversial and under debate. It is known that vitamin D deficiency (25(OH)D < 20 ng/mL) and insufficiency (25(OH)D 20–29 ng/mL) are highly prevalent, affecting over 80% of HD patients, and should be corrected [2,5,6]. However, the prevailing opinion is that PTH lowering with cholecalciferol is unproven and clinically insignificant. Our recent study showed that correction of 25(OH)D > 30 ng/mL with high doses of cholecalciferol did not influence PTH levels and only a few patients achieved a concentration of 1,25(OH)_2_D above the lower limit of the reference range [7]. There are some indications that cholecalciferol may even be useful in CKD-MBD in HD patients; however, the therapy must result in significantly higher levels of 25(OH)D [8]. In the present study, we evaluated the influence of cholecalciferol treatment targeting a high normal level of 25(OH)D on biochemical markers of CKD-MBD, including 1,25(OH)_2_D and 1–84 PTH. The secondary goal was to establish a maintenance dose of cholecalciferol to keep the 25(OH)D level in the range of 50–75 ng/mL.

## 2. Materials and Methods

### 2.1. Study Design

This was an observational prospective study consisting of three phases in a cohort of CKD stage 5D patients from a single HD center. In the therapeutic phase of the study, patients with an initial 25(OH)D serum concentration between 30–50 ng/mL were administered cholecalciferol until they reached the target 25(OH)D level > 75 ng/mL, but not longer than for 24 weeks. The treatment efficacy was measured by change in 1,25(OH)_2_D and 1–84 PTH levels. In detail, the 1,25(OH)_2_D and 1–84 PTH levels when 25(OH)D reached 30 ng/mL (T0) were compared with the 1,25(OH)_2_D and 1–84 PTH levels when 25(OH)D reached 75 ng/mL, or when the highest 25(OH)D was achieved after 24 weeks of cholecalciferol treatment (Tmax). In the maintenance phase (after reaching Tmax), the cholecalciferol dose was adjusted depending on 25(OH)D levels to hold the levels between 50–75 ng/mL. The maintenance dose for a single patient was determined by confirming that, during its use, 25(OH)D concentrations in three consecutive tests remained in the 50–75 range.

### 2.2. Patients

The study was a continuation of the observational study described earlier and included the same group of patients treated with cholecalciferol [7]. They qualified for the present study when 25(OH)D concentration exceeded 30 ng/mL. The exclusion criteria included the following: inadequately controlled SHP (PTH > 800 pg/mL), treatment with calcimimetics or active forms of vitamin D3, parathyroidectomy, bilateral nephrectomy, treatment with corticosteroids, and dialysis time of less than 3 months.

### 2.3. Therapeutic and Maintenance Dose Management

**Figure 1:**
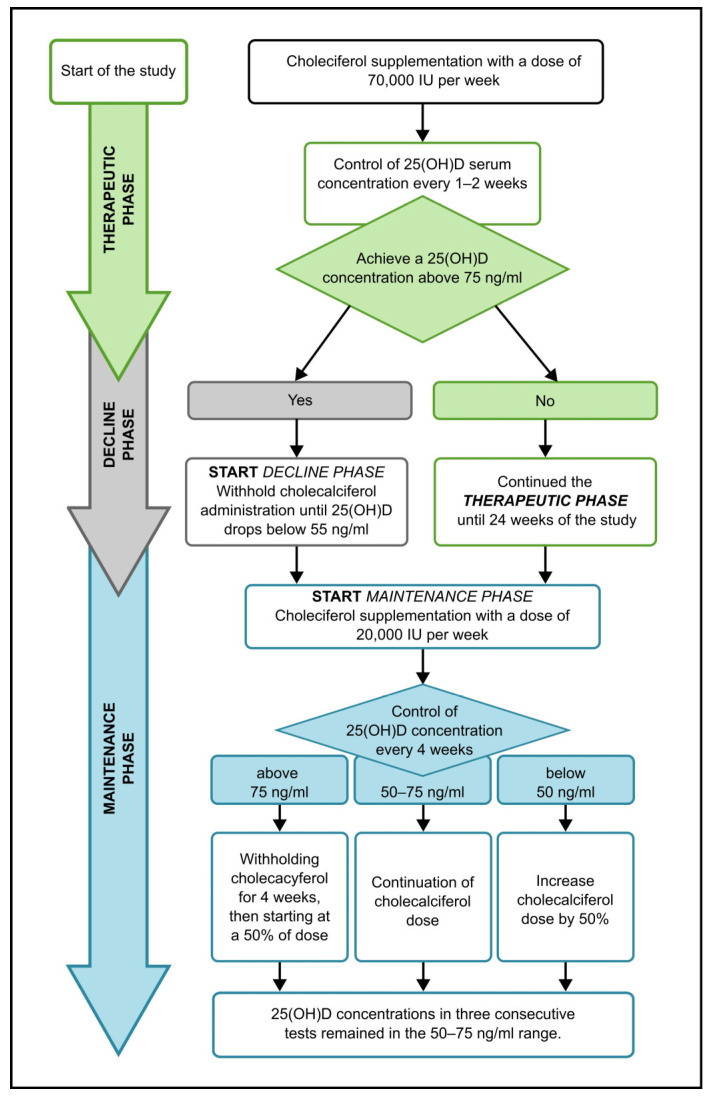
Study flow chart. All patients upon study initiation entered the therapeutic phase, during which they supplemented cholecalciferol at a dose of 70,000 IU/week. Serum 25(OH)D levels were monitored weekly, and if 25(OH)D concentrations reached above 75 ng/mL, patients transitioned to the decline phase, during which cholecalciferol administration was suspended. The reintroduction of cholecalciferol occurred upon 25(OH)D levels falling below 55 ng/mL and completed the decline phase and the beginning of the maintenance phase. Patients who did not achieve a 25(OH)D concentration above 75 ng/mL by week 24 of the study immediately transitioned to the maintenance phase (without the decline phase), during which they were administered 20,000 IU cholecalciferol/week. In the maintenance phase, 25(OH)D levels were monitored every 4 weeks, and the dose was adjusted to maintain levels within the range of 50–75 ng/mL.

The patients received a therapeutic dose of cholecalciferol at 70,000 IU per week (20,000 IU + 20,000 IU + 30,000 IU per week) administered immediately after each dialysis session. Observation continued until the target 25(OH)D level exceeded 75 ng/mL, but not longer than for 24 weeks. In the maintenance phase, the cholecalciferol dose (20,000–40,000 IU after the first dialysis session in the week) was adjusted according to 25(OH)D level to keep it in a range of 50–75 ng/mL. Figure 1 shows how cholecalciferol treatment was administered and adjusted.

### 2.4. Laboratory Measurements

During the therapeutic phase, concentrations of 25(OH)D, 1,25(OH)_2_D, calcium, phosphate, 1–84 PTH were monitored at intervals of 1–2 weeks, and then every 4 weeks during the maintenance phase. Prior to a dialysis session, a 5 mL blood sample was taken, collected in a standard tube, and allowed to clot at room temperature. Following this, the blood was centrifuged and preserved at a temperature of −20 °C.

#### 2.4.1. 25(OH)D and 1,25(OH)_2_D

The levels of serum 1,25(OH)_2_D and 25(OH)D were determined using the chemiluminescent immunoassay technique. The LIAISON^®^ XL 1,25 Dihydroxyvitamin D and LIAISON^®^ 25 OH Vitamin D TOTAL Assay, both by DiaSorin, Saluggia, Italy, were used for this purpose. The laboratory’s normal range for 1,25(OH)_2_D is 25–86.5 pg/mL, while for 25(OH)D it is 30–80 ng/mL. The 95% Reference Interval established the following values for 1,25(OH)_2_D: median 47.8 pg/mL; observed range 2.5th to 97.5th percentile 19.9–79.3 pg/mL.

#### 2.4.2. 1–84 PTH

1–84 PTH was quantified using a chemiluminescent assay, DiaSorin LIAISON^®^1-84 PTH, with a laboratory normal range of 4.6–58.1 pg/mL. 1–84 PTH has 0% cross-reactivity to 7–84 and other fragments and is 100% specific to 1–84 PTH.

#### 2.4.3. Calcium and Phosphorus

Total serum calcium and phosphorus were measured by routine colorimetric method (Abbott GmbH & Co., Wiesbaden, Germany). The assessment of the safety profile included monitoring episodes of hypercalcemia (following the KDIGO recommendations defined as a rise in calcium levels of more than 10.5 mg/dL) and hyperphosphatemia (defined as phosphorus levels of more than 5.5 mg/dL).

### 2.5. Statistical Analysis

Continuous data are reported as means (±standard deviation, SD) for normally distributed variables or medians (interquartile ranges, IQR) for non-normally distributed variables. Categorical data are reported as percentages of the total. The Shapiro–Wilk test was used to determine the distribution of continuous variables. The comparisons of paired variables were performed using Student’s *t*-test or a Wilcoxon signed-rank test, where appropriate. In strata analyses, the analysis of variance (ANOVA) was performed with Bonferroni corrections for paired comparisons. χ^2^ test or Pearson’s χ^2^ test was used for independent categorical variables, while for dependent variables (repeated measures or paired data), the McNemar test was applied. The Pearson correlation coefficient or Spearman rank correlation coefficient was calculated to measure an association between variables as appropriate. Multivariate linear mixed-effect regression model was used to assess independent correlation between the repeatedly tested variables—parameters of calcium and phosphate metabolism. The model included variables that showed relationships in univariate analyses, i.e., 25(OH)D, 1,25(OH)_2_D and 1–84 PTH, and arbitrarily selected factors with a potential confounding effect, i.e., age, gender, and BMI. The missing data were singular and occurred at random; hence, simple imputation techniques were applied as necessary to address these gaps. The significance of the model parameters was evaluated utilizing the Wald Chi-squared statistic. Two-sided *p* < 0.05 was considered to be statistically significant. The statistical analysis was performed using the program Statistica 13.3 (TIBCO Software Inc.; Palo Alto, CA, USA).

## 3. Results

### 3.1. Characteristics of the Study Group

The study included a total of 22 patients, comprising 16 men (73%) and 6 women (27%), with an average age of 72.5 ± 13.33 years. The median HD vintage at baseline was 25 (12–56) months. Coronary artery disease was diagnosed in 11 out of 22 patients (50%), heart failure in 11 out of 22 (50%), and peripheral arterial disease in 8 out of 22 (36.36%). The Charlson Comorbidity Index was 8 points (2–14), and the Body Mass Index was 25.27 (22.50–28.76) kg/m^2^. Their dialysis treatment protocols were as follows: high-flux HD was performed in eighteen patients, extended HD (Theranova, Baxter^®^, Deerfield, IL, USA) was received by two patients, one patient underwent online hemodiafiltration (HDF) in predilution mode, and one patient online HDF in post-dilution mode. High-flux HD and online HDF sessions were performed with FX dialyzers (Helixone-Fresenius^®^, Bad Homburg vor der Höhe, Germany). The Kt/V coefficient in the patients was 1.66 ± 0.21, with a median dialysis time of 720 (720–765) minutes per week. The dialysate Ca concentration used was 1.25 mmol/L for 21 patients and 1.5 mmol/L for 1 patient. Throughout the study, dialysis protocol and doses of medications affecting calcium and phosphate metabolism, such as phosphate binders, persisted without changes. Due to exclusion criteria, the patients were not administered active forms or analogs of vitamin D or calcimimetics. A detailed description of the study group has been provided previously [7].

### 3.2. Laboratory Measurements

#### 3.2.1. 25(OH)D

During the therapeutic phase, a statistically significant increase in 25(OH)D concentration was observed (*p* < 0.005) (Table 1). The target 25(OH)D concentration above 75 ng/mL was achieved in 73% of patients (16/22). The median time required to reach target concentration in these patients was 7.5 weeks (4.0–20.0), with a median total dose of cholecalciferol of 525,000 IU (280,000–1,400,000). A concentration of 25(OH)D above 50 ng/mL was achieved in all patients. The median time to reach this concentration was 6 weeks (5.5–9). One patient withdrew study consent during the therapeutic phase.

SI conversion factors: to convert calcium to mmol/L, multiply by 0.25; phosphorus to mmol/L, by 0.323; 1–84 PTH to pmol/L, by 0.106; 25(OH)D to nmol/L, by 2.496; 1,25(OH)_2_D to pmol/L, by 2.4. Abbreviations: CKD-MBD, Chronic Kidney Disease—Mineral and Bone Disorder; 1–84 PTH, parathyroid hormone 1–84; 25(OH)D, 25-hydroxycholecalciferol, calcidiol; 1,25(OH)_2_D, 1,25-dihydroxycholecalciferol, calcitriol.

#### 3.2.2. 1,25(OH)_2_D

**Figure 2:**
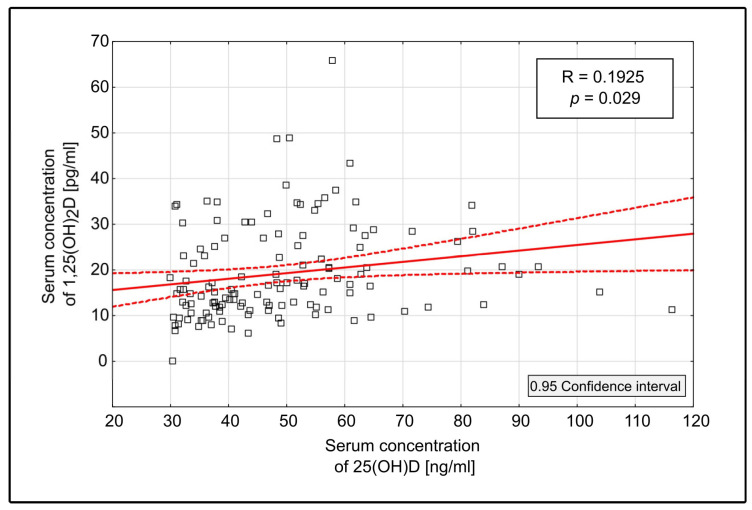
Correlation between serum concentration of 25(OH)D and 1,25(OH)_2_D throughout the entire study period. The solid line represents the line of best fit, while the dashed lines indicate the confidence interval lines.

During the therapeutic phase, a statistically significant increase in the 1,25(OH)_2_D concentration was noted (*p* = 0.002). Detailed results are presented in Table 1. At the commencement of the study (T0), 9% (2/22) of the patients showed a normal 1,25(OH)_2_D serum concentration. A total of 50% (11/22) of the patients reached a normal 1,25(OH)_2_D concentration at the end of the therapeutic phase (Tmax) (*p* = 0.03). There was a significant statistical correlation between the concentration of 25(OH)D and 1,25(OH)_2_D (R = 0.1925, *p* = 0.029), as illustrated in Figure 2. This analysis was performed using 129 available pairs of measurements (25(OH)D and 1,25(OH)_2_D) in 22 patients during all phases of the study.

In the secondary strata analysis performed using all available pairs of 25(OH)D-1,25(OH)_2_D measurements during all phases of the study, the level of 1,25(OH)_2_D varied depending on the obtained ranges of 25(OH)D concentrations (ANOVA: *p* < 0.001). The concentration of 1,25(OH)_2_D was significantly higher in patients with 25(OH)D levels in the range of 50–75 ng/mL (*p* < 0.005) relative to those with levels in the range of 30–49.9 ng/mL. This difference was insignificant within the 30–49.9 ng/mL range and above 75 ng/mL (*p* = 0.36). There were no differences in 1,25(OH)_2_D concentrations between patients with 25(OH)D levels between 50–75 ng/mL and above 75 ng/mL (*p* = 0.097). Similarly, the percentage of patients who achieved normal 1,25(OH)_2_D levels significantly increased depending on the obtained 25(OH)D concentrations. The proportion of normal concentrations of 1,25(OH)_2_D was higher in patients with 25(OH)D concentrations of 50 ng/mL and above (*p* = 0.0079) compared to those with levels in the range of 30–49.9 ng/mL. There were no differences between subgroups with 25(OH)D concentrations between 50–75 ng/mL and above 75 ng/mL (*p* = 0.06). Detailed results are presented in Table 2.

#### 3.2.3. 1–84 PTH

**Figure 3:**
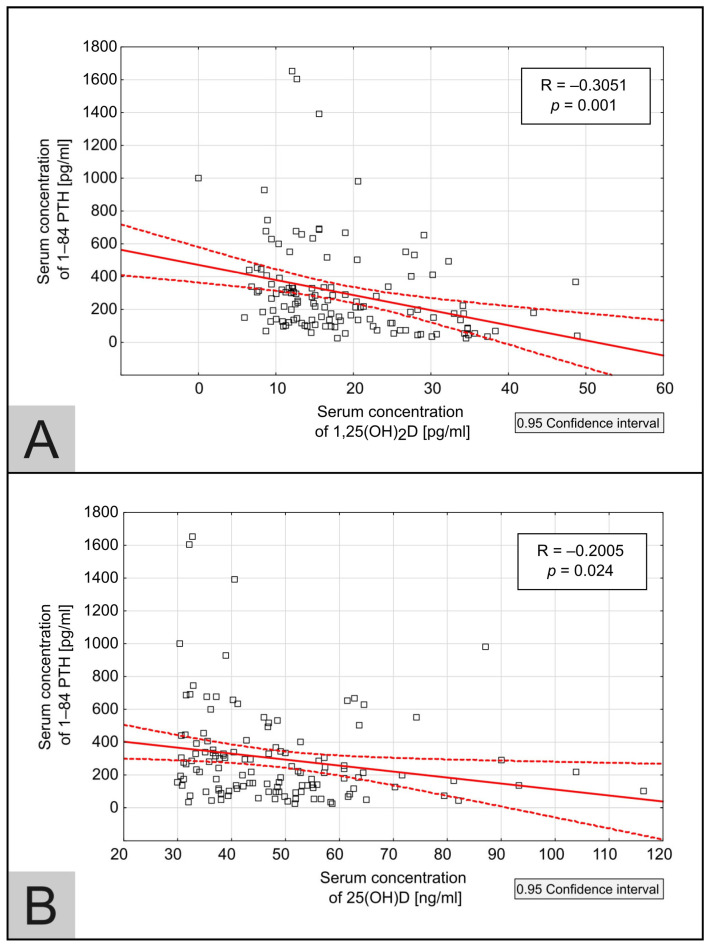
A scatter plot of the correlation between the serum concentration of 1–84 PTH and 1,25(OH)_2_D (**A**) and between 1–84 PTH and 25(OH)D (**B**) throughout the entire study period. The correlation coefficient (R) and *p*-value (*p*) are provided on the graph. The solid line represents the line of best fit, while the dashed lines indicate the confidence interval lines.

During the treatment phase, a statistically significant decrease in 1–84 PTH concentration was observed (*p* < 0.005). Detailed results are presented in Table 1. There was a significant statistical correlation between 25(OH)D and 1–84 PTH (R = −0.2005, *p* = 0.024) and between 1,25(OH)_2_D and 1–84 PTH (R = −0.3051, *p* = 0.001) as illustrated in Figure 3. The analysis was performed using 133 available measurements in 22 patients throughout the entire study period.

#### 3.2.4. Independent Associations Between 25(OH)D, 1,25(OH)_2_D, 1–84 PTH

In a multivariate mixed-effect regression model for 133 repeated measurements, including 25(OH)D, 1,25(OH)_2_D, 1–84 PTH, age, sex, and BMI, we found significant association between 25(OH)D and 1,25(OH)_2_D (β = 0.32, z = 3.64, SE 0.098; *p* < 0.001) and a negative association between 25 (OH)D and 1–84 PTH (β = −0.021, z = −3.07, SE 0.007; *p* = 0.002). The model shows good fit and statistical significance confirmed by the Wald Chi-squared test (χ^2^ = 51.9 *p* < 0.001).

#### 3.2.5. Calcium

During the study, no episodes of hypercalcemia (over 10.5 mg/dL) were detected. The ranges of calcium serum concentration in the therapeutic phase are presented in Table 1.

#### 3.2.6. Phosphorus

There was no significant change in phosphorus concentration during the study. The detailed results are presented in Table 1. Hyperphosphatemia was observed in 50% of the patients (11/22) at the beginning of the study (T0) and in 45% (10/22) at the end of the therapeutic phase (Tmax).

### 3.3. The Maintenance Cholecalciferol Dose Adjustment

**Figure 4:**
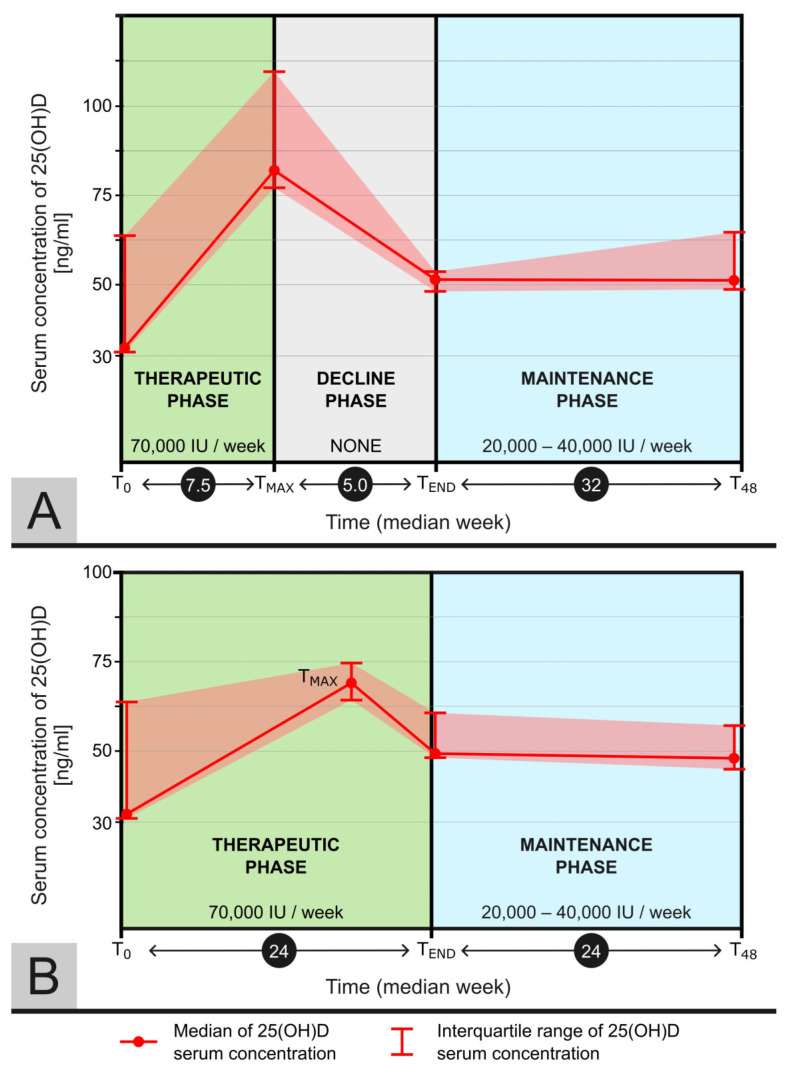
Median concentration of 25(OH)D during the study at 4 time points: at the beginning of the study (T0), at the point of the highest achieved concentration (Tmax), at the end of the decline phase (Tend), and at the end of the study (T48). The Tmax point for patients who achieved a concentration of 25(OH)D above 75 ng/mL (**A**) was equivalent to the end of the therapeutic phase and the beginning of the decline phase. For patients who did not achieve a concentration of 25(OH)D above 75 ng/mL (**B**) within 24 weeks of using cholecalciferol at a dose of 70,000 IU per week, they immediately transitioned (without a decline phase) to the maintenance phase. The doses of cholecalciferol used in each phase are provided below the phase names.

In patients who achieved target 25(OH)D concentrations above 75 ng/mL (16 patients), cholecalciferol administration was discontinued until level 25(OH)D dropped below 55 ng/dL (decline phase), as shown in Figure 4A. The median duration of treatment interruption was 5 weeks (4–8). Notably, one patient (6.25%) required as many as 22 weeks of treatment interruption. Among the remaining five patients (those who have not reached the 25(OH)D target > 75 ng/mL), the maintenance phase was initiated immediately after the therapeutic phase (without the decline phase), presented in Figure 4B.

The median duration of the maintenance treatment phase was 30 weeks (24–32). In 47.6% of the patients (10/21), a weekly dose of 20,000 IU/week of cholecalciferol was sufficient to maintain a stable level of 25(OH)D between 50–75 ng/mL. For 42.9% of the patients (9/21), a dose of 40,000 IU/week was required, while the remaining 9.5% (2/21) required an intermediate dose of cholecalciferol of 30,000 IU/week. The median maintenance dose was 30,000 IU/week (20,000–40,000 IU).

## 4. Discussion

This study aimed to evaluate the impact of cholecalciferol treatment targeting a high normal concentration of 25(OH)D on biochemical markers of CKD-MBD, primarily 1,25(OH)_2_D and 1–84 PTH in hemodialysis patients. The presented results show, first of all, a significant improvement in vitamin D status, as evidenced by the increase in serum concentrations of 25(OH)D and 1,25(OH)_2_D. In the study group, the majority reached the target 25(OH)D concentration of over 75 ng/mL. This was achieved within a median time of 7.5 weeks, with a median total cholecalciferol dose of 525,000 IU. All participants attained levels above 50 ng/mL within a median timeframe of 6 weeks. Our previous study, as well as many other reports focused on normalization of 25(OH)D, that was achieved in 57–100% patients depending on the doses of cholecalciferol and the treatment time [7,9,10,11,12,13,14,15]. Higher 25(OH)D levels exceeding 50 ng/mL were reached by Wasse et al. [14].

Furthermore, the rise in the 25(OH)D level was followed by a significant increase in 1,25(OH)_2_D levels with the notable rise of the percentage of patients achieving normal serum concentrations. This is consistent with the understanding that vitamin D metabolism is partially active in hemodialysis patients and that cholecalciferol supplementation is sensible and can contribute to the normalization of 1,25(OH)_2_D levels in this patient population. The significant rise in 1,25(OH)_2_D levels in HD patients on cholecalciferol supplementation has already been reported; however, the data on the percentage of patients reaching the normal values were not included [9,10,11,12,13,14,15]. Our study shows that the level of active vitamin D, and the percentage of patients achieving normalization of its level, varied significantly, with different ranges of 25(OH)D concentrations. It was observed that the attainment of a 25(OH)D concentration above 50 ng/mL was associated with significantly higher levels of 1,25(OH)_2_D and the percentage of patients with a normal concentration of 1,25(OH)_2_D. Correspondingly, Wasse et al. reported on the 25(OH)D levels of 52.4 ng/mL and 1,25(OH)_2_D 29.3 pg/mL at the end of the study [14]. However, no significant impact on active vitamin D status was observed with further elevation of 25(OH)D above 75 ng/mL in our study. The correlation between 25(OH)D and 1,25(OH)_2_D concentrations was statistically significant; however, a low-degree association between the variables (R = 0.1925, *p* = 0.029) suggests a notable but modest relationship and indicates that other factors may also play a role in determining 1,25(OH)_2_D levels.

A statistically significant decrease in 1–84 PTH levels was also observed in the treatment phase of the study, which is clinically relevant as SHP is a crucial manifestation of CKD-MBD. The significant negative correlations between 25(OH)D and 1–84 PTH, as well as 1,25(OH)_2_D and 1–84 PTH levels, suggest that adequate cholecalciferol supplementation with adequate 25(OH)D and 1,25(OH)_2_D levels may play an important role in mitigating parathyroid gland overactivity in the HD patients population. The achieved results indicate not only the impact on prevention of SHP, but also suggest that treatment with cholecalciferol may be beneficial in the management of SHP in HD patients. The data from various studies present conflicting results. Omrani et al. showed equal effectiveness of cholecalciferol and calcitriol in reducing PTH levels in HD patients, and it is worth noticing that in the cholecalciferol group, an increase in serum 25(OH)D levels to 62.96 ng/mL was observed [8]. Some other research indicates that a decrease in PTH was observed when 25(OH)D levels reached approximately 40 ng/mL [15,16,17]; other studies did not show any impact on PTH, even when the concentration of 25(OH)D exceeded 50 ng/mL [14]. Similarly, the decrease in PTH levels was reported even if 1,25(OH)_2_D levels did not reach the lower limit of the laboratory norms (18.75 pg/mL and 20.5 pg/mL, respectively [15,16]). On the other hand, the normal 1,25(OH)_2_D levels of 29.3 pg/mL and 40.08 pg/mL did not result in a PTH drop [14,18]. As the local production of active vitamin D in cells occurs, and also because 25(OH)D may directly activate the vitamin D receptor, serum 1,25(OH)_2_D levels may not simply reflect the clinical effectiveness of cholecalciferol therapy on parathyroids [19]. Recently, extended-release calcifediol (25(OH)D) for SHP has been gaining interest. When used in pre-dialysis CKD patients, it showed that PTH was suppressed as 25(OH)D levels reached at least 50.8 ng/mL; moreover, elevation to 92.5 ng/mL was not associated with adverse events, indicating that targets for vitamin D repletion therapy in CKD might be higher [20].

This study also highlights the importance of individualized dosing during the maintenance phase to prevent relapse into vitamin D deficiency. The findings suggest that a weekly dose of 20,000 to 40,000 IU of cholecalciferol is effective for most patients, with a median maintenance dose of 30,000 IU/week. The maintenance of stable calcium and levels within the normal range throughout the study indicates that the cholecalciferol dosing regimen was safe and did not lead to hypercalcemia or significant changes in phosphorus homeostasis, even though a significant increase in active vitamin D levels was achieved with potential influence on calcium and phosphorous gut absorption. This contrasts with the studies with active vitamin D supplementation [21,22].

A key strength of the study was the supervised administration of cholecalciferol at the HD unit, ensuring high compliance. Furthermore, the patients were not taking the active form of vitamin D, its analogues, or calcimimetics for several months before and during the study; therefore, the effects can be entirely ascribed to the administration of cholecalciferol. Lastly, our study offers real-world data, presenting the diversity of a typical dialysis unit. However, the unit was applying high-efficiency dialysis with methods tailored to each patient. There are also several strong clinical points of the study. It underscores the need for personalized dosing regimens of cholecalciferol to achieve and maintain optimal vitamin D levels in HD patients, although average total treatment and maintenance cholecalciferol doses may help in applying research results to clinical practice. Regular laboratory monitoring seems to be crucial to adjust supplementation doses and prevent both deficiency and toxicity. The significant decrease in PTH levels implies that adequate vitamin D supplementation could be a key component in the management of SHP in HD patients and may reduce the need for other interventions. The absence of hypercalcemia episodes and no deterioration in hyperphosphatemia indicate that cholecalciferol supplementation is safe when appropriately monitored. It supports the broader implementation of cholecalciferol supplementation protocols in HD patients.

Several limitations should be considered when interpreting the study’s results. The main limitation was the small number of participants; however, this resulted from strict exclusion criteria. The study group consisted mostly of older men, who are the most common type of patient encountered in dialysis centers. The study was designed as a single-center preliminary observational study without a control group. We admit that this makes it difficult to establish a causal relationship between cholecalciferol supplementation and the observed outcomes and limits the generalizability of the findings to the broader HD population. Finally, the study focused on biochemical parameters without assessing clinical outcomes such as bone mineral density, fracture rates, or cardiovascular events, and without long-term observation. Future research, especially concerning the sustainability of vitamin D levels and the long-term effects on bone and cardiovascular health, is vital to build upon these findings.

## 5. Conclusions

In conclusion, this study provides evidence supporting the use of cholecalciferol to correct biochemical markers of CKD-MBD in hemodialysis patients. The positive outcomes observed in 25(OH)D and 1,25(OH)_2_ levels, along with the management of PTH, calcium, and phosphorus levels, underscore the potential benefits of vitamin D supplementation in this vulnerable group. The individualized and monitored approach to supplementation could potentially reduce the burden of complications associated with CKD-MBD. Future research should focus on long-term outcomes, including the impact of high-dose cholecalciferol supplementation on bone mineral density and cardiovascular health.

## Figures and Tables

**Table 1:** CKD-MBD biochemical parameters at baseline T0 and Tmax (at the time of reaching a concentration 25(OH)D above 75 ng/dL or, if this has not occurred, at the time of achieving the highest level of 25(OH)D).

Parameters	Serum Concentration at T0	Serum Concentration at Tmax	*p*-Value
Calcium [mg/dL]	9.04 (7.8–8.8)	9.1 (9.84–8.3)	0.23
Phosphorus [mg/dL]	4.95 (3.9–5.7)	5.05 (3.9–5.7)	0.93
1–84 PTH [pg/mL]	416 (275–685)	214.0 (215–636)	<0.005
25(OH)D [ng/mL]	32.9 (31.6–35.2)	81.05 (69–90.1)	<0.005
1,25(OH)_2_D [pg/mL]	12.05 (8.9–9.3)	22.8 (16.6–34.4)	<0.005

The data are presented as the median and interquartile range (IQR), with statistical analysis conducted using the Wilcoxon signed-rank test between groups (T0 vs. Tmax). *p* values < 0.05 were considered significant.

**Table 2:** Strata analysis of 1,25(OH)_2_D levels depending on 25(OH)D concentration ranges obtained.

25(OH)D Range [ng/mL]	1,25(OH)_2_ D [pg/mL]	1,25(OH)_2_D in Normal Range
30–49.9 (*n* = 78)	13.95 (10.5–21.4)	15.39%
50–75 (*n* = 38)	20.45 (16.3–33.0)	42.11%
over 75 (*n* = 13)	19.70 (12.3–26.1)	46.15%

The data are presented as the median and interquartile range (IQR). This analysis was performed using 129 available pairs of measurements (25(OH)D and 1,25(OH)_2_D) in 22 patients during all phases of the study. The 1,25(OH)_2_D in normal range was defined as the percentage of results within the laboratory’s normal range for 1,25(OH)_2_D, which was 25–86.5 pg/mL. Abbreviations: 25(OH)D, 25-hydroxycholecalciferol, calcidiol; 1,25(OH)_2_D, 1,25-dihydroxycholecalciferol, calcitriol.

## Data Availability

Data available upon request.
